# Blubber transcriptome responses to repeated ACTH administration in a marine mammal

**DOI:** 10.1038/s41598-019-39089-2

**Published:** 2019-02-25

**Authors:** Jared S. Deyarmin, Molly C. McCormley, Cory D. Champagne, Alicia P. Stephan, Laura Pujade Busqueta, Daniel E. Crocker, Dorian S. Houser, Jane I. Khudyakov

**Affiliations:** 1 Department of Biological Sciences, University of the Pacific, Stockton, CA 95211 USA; 2 Conservation and Biological Research Program, National Marine Mammal Foundation, San Diego, CA 92106 USA; 3 Biology Department, Sonoma State University, Rohnert Park, CA 94928 USA

## Abstract

Chronic physiological stress impacts animal fitness by catabolizing metabolic stores and suppressing reproduction. This can be especially deleterious for capital breeding carnivores such as marine mammals, with potential for ecosystem-wide effects. However, the impacts and indicators of chronic stress in animals are currently poorly understood. To identify downstream mediators of repeated stress responses in marine mammals, we administered adrenocorticotropic hormone (ACTH) once daily for four days to free-ranging juvenile northern elephant seals (*Mirounga angustirostris*) to stimulate endogenous corticosteroid release, and compared blubber tissue transcriptome responses to the first and fourth ACTH administrations. Gene expression profiles were distinct between blubber responses to single and repeated ACTH administration, despite similarities in circulating cortisol profiles. We identified 61 and 12 genes that were differentially expressed (DEGs) in response to the first ACTH and fourth administrations, respectively, 24 DEGs between the first and fourth pre-ACTH samples, and 12 DEGs between ACTH response samples from the first and fourth days. Annotated DEGs were associated with functions in redox and lipid homeostasis, suggesting potential negative impacts of repeated stress on capital breeding, diving mammals. DEGs identified in this study are potential markers of repeated stress in marine mammals, which may not be detectable by endocrine profiles alone.

## Introduction

Increasing anthropogenic activity has been correlated with loss of biodiversity in many ecosystems^1^. Consequences of anthropogenic disturbance, such as declines in apex predator populations, are causing detrimental shifts in food web dynamics^2^. For example, the collapse of large marine carnivore populations has been correlated with altered trophic structure and decline of commercially valuable fish stocks^3^. Marine mammal populations are threatened by noise pollution, ship strikes, fisheries competition and bycatch, prey declines, habitat loss, and climate change^4–7^. In addition to direct effects on survival, anthropogenic disturbance may impact marine mammal health, fitness, and population persistence by causing physiological stress.

Stress response pathways maintain homeostasis and mediate the relationship between an animal and its external environment^8^. The mammalian stress response is mediated by the hypothalamic-pituitary-adrenal (HPA) axis^9^. In response to psychological or physiological stress, corticotropin-releasing hormone (CRH) is released by the hypothalamus, triggering the release of adrenocorticotropic hormone (ACTH) by the pituitary gland. This stimulates synthesis of corticosteroids (glucocorticoids and mineralocorticoids) by the adrenal glands. Stress studies in terrestrial mammals have primarly focused on downstream effects of glucocorticoids (e.g. cortisol) rather than mineralocorticoids, but a number of studies have shown that the mineralocorticoid aldosterone is an important component of stress responses in marine mammals^10–12^. Corticosteroids circulate in blood complexed with binding globulins (e.g. corticosteroid binding globulin); free hormones exert biological effects by binding to cell surface and intracellular receptors – glucocorticoid receptor, GR, and mineralocorticoid receptor, MR – in target tissues (e.g. adipose, muscle, and liver). Cell surface receptors alter signaling pathways, while intracellular hormone-receptor complexes translocate to the nucleus, bind glucocorticoid and mineralocoid response elements of target genes, and impact gene transcription^13^. Altered expression of target genes, such as those encoding metabolic enzymes and hormones, promotes the physiological adjustments required to overcome the stressor. These adjustments include mobilization of glucose and lipid stores to meet increased energy demands and transient suppression of energetically expensive processes, such as reproduction, growth, and immune responses^9^. While short-term (acute) stress responses are adaptive^14–16^, repeated (chronic) stress exposure may deplete energy stores and impair immunity and reproduction, reducing fitness. This could be detrimental to threatened animal populations that already experience nutritional stress and low fecundity, such as some marine mammals^17^. However, little is known about the indicators and downstream effects of repeated stress in wildlife, hindering the ability of conservation biologists to identify stressed animals and predict the effects of repeated stress on population stability^18,19^.

Marine mammals rely on endogenous energy reserves to support life history stages characterized by high energy expenditure and nutrient limitation. For example, semiaquatic marine mammals (e.g. pinnipeds – seals and sea lions) reproduce on land using a capital breeding strategy, in which animals rely solely on energy stores to fuel metabolic demands of reproduction^20,21^. Many fully aquatic marine mammals (e.g. cetaceans) are also capital breeders that migrate long distances between foraging and breeding grounds^22^. Both examples necessitate energy accumulation and storage during feeding and energy mobilization during fasting, a process that occurs for several weeks to several months as part of marine mammal life history. The main energy depot in cetaceans and pinnipeds is blubber, a specialized type of subcutaneous adipose tissue that plays significant roles in metabolism (similarly to white adipose tissue, WAT, in other mammals) and thermoregulation. Blubber is vertically stratified into at least two layers: an outer layer used for thermoregulation, and an inner, metabolically active layer^23^. WAT is a major target of stress hormones in mammals^24^ and studies in marine mammals have shown that acute HPA axis activation mobilizes stored lipids^10,25^. It is plausible that chronic or repeated stress would deregulate fasting metabolism and deplete lipid stores, among other impacts, limiting the ability of capital breeders to meet the energetic demands of reproduction and leading to declines in fitness.

In this study, we evaluated the effects of repeated HPA axis activation on blubber gene expression in a fasting-adapted marine mammal, the northern elephant seal (*Mirounga angustirostris*). Elephant seals are an established marine mammal study system in which variation in baseline hormone levels^26^, responses to acute ACTH administration^14,25,27,28^, and metabolic adjustments during fasting^29,30^ have been described. In addition, anesthesia procedures have been shown to minimize handling stress in this species, enabling measurements of true baseline hormone and gene expression levels^25^. We simulated repeated physiological stress exposure by administering synthetic ACTH to juvenile elephant seals once daily for 4 days, inducing significant elevations in corticosteroid levels that we described in a previous publication^12^. To evaluate downstream mechanisms by which repeated HPA axis activation may impact metabolic homeostasis, we compared transcriptome responses of elephant seal blubber tissue to the first and last ACTH administration by RNA sequencing (RNAseq). We identified 27 genes that were differentially expressed in blubber during the response to the fourth versus the first ACTH administration, many of which were associated with lipid storage and mobilization. These genes are potential biomarkers for discriminating between acute and repeated ACTH responses in marine mammals, which otherwise cannot be distinguished by cortisol profiles alone.

## Materials and Methods

### Study animals and experimental design

**Figure 1:**
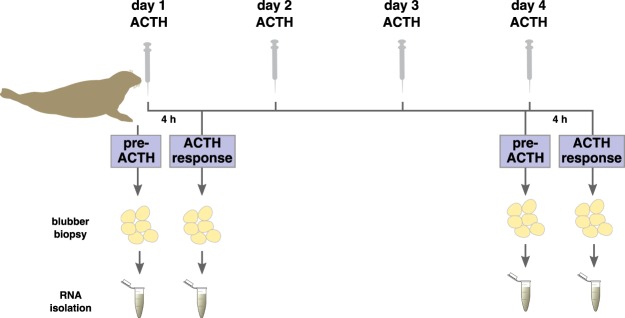
Repeated ACTH administration experiment (described in McCormley *et al*.^12^) and blubber tissue sampling design. ACTH was administered to juvenile elephant seals once every 24 hours for 4 days. Blubber biopsies were collected immediately before ACTH administration (“pre-ACTH”) and 4 hours after ACTH administration (“ACTH response”) on the first and fourth days. RNA isolated from blubber tissue was used for transcriptome sequencing. Seal (color modified, from https://pixabay.com), syringe (from https://pixabay.com), adipose (enhanced contrast, from https://openclipart.org), and microcentrifuge tube (from http://www.clker.com) images were obtained under CC0 1.0 Universal: CC0 1.0 Public Domain Dedication. License: https://creativecommons.org/publicdomain/zero/1.0.

Juvenile (0.8-year old) northern elephant seals (*Mirounga angustirostris*) were sampled at Año Nuevo State Reserve (San Mateo County, CA) in Aug.–Nov. 2016. All animal handling protocols were approved by Sonoma State University and University of the Pacific Institutional Animal Care and Use Committees (IACUC), Department of the Navy Bureau of Medicine and Surgery (BUMED), and were conducted under National Oceanic and Atmospheric Administration (NOAA) Fisheries Permit No. 19108. All procedures involving animals were conducted in accordance with the relevant guidelines and regulations of IACUC and BUMED protocols and the NOAA permit. Repeated stress was simulated by administering ACTH to each study animal once every 24 hours for 4 consecutive days. Blood and blubber samples were collected before ACTH administration (“pre-ACTH”) and 4 hours after ACTH administration (“ACTH response”) on the first and fourth days (Fig. 1). Details of the experimental manipulation and hormone response have been reported previously^12^. In summary, ACTH administration induced significant elevation in circulating cortisol and aldosterone levels and decline in total triiodothyronine (T3) levels. Cortisol levels recovered to baseline within 24 hours of each ACTH administration and cortisol responses to ACTH did not vary in magnitude between the first and fourth administrations. However, aldosterone responses to ACTH were higher on day 4 than on day 1 and did not recover to baseline between administrations (for details, see McCormley *et al*.^12^). Four of the 7 animals that participated in the repeated ACTH administration experiment described in McCormley *et al*.^12^ (seals 2, 4, 6, and 7) were selected for the current study based on amounts of sequencing quality RNA that could be obtained from blubber samples.

### ACTH administration and blubber sampling

Study animals were chemically immobilized and blood samples were collected as previously described^12^. Blubber samples (“day 1 pre-ACTH”) were collected from the posterior flank region of each animal using a 6.0 mm diameter biopsy punch (Miltex, USA). The inner half (closest to musculature) of each biopsy sample was isolated, minced, and placed in cryovials containing RNA*later*™ Stabilization Solution (1.5 mL per ~300 mg tissue; Invitrogen, USA). Samples were incubated for 24 hours at 4 °C, after which RNA*later*™ solution was removed and samples were stored at −80 °C until RNA isolation. After baseline sampling, animals received 20 U of corticotropin LA gel (Wedgewood Pharmacy, USA) via intramuscular injection (mass specific dose: 0.17 ± 0.02 U/kg). A paired set of blubber and blood samples was collected 4 hours after ACTH administration (“day 1 ACTH response”); biopsies were collected from the contralateral side of the animal to the ACTH injection. On the second and third days of the experiment, ACTH was administered approximately 24 hours after the injection from the previous day, but no post-ACTH sampling was conducted. On the fourth day, pre-ACTH sampling (“day 4 pre-ACTH”), ACTH administration, and post-ACTH sampling (“day 4 ACTH response”) were conducted as described for the first day.

### RNA isolation

Blubber samples were homogenized by bead beating in Qiazol (Qiagen, USA; ~100 mg tissue per 0.5 mL) using Bullet Blender Storm 24 (Next Advance, USA; Speed 12, two 2-minute cycles). After bead beating, another 0.5 mL of Qiazol was added to each sample and incubated for 5 minutes at room temperature with occasional vortexing. Lysates were further disrupted using QiaShredder tubes (Qiagen, USA) to shear genomic DNA and centrifuged for 15 minutes to separate lipids and cellular components. RNA was isolated from homogenates using the Lipid RNeasy Tissue Kit (Qiagen, USA) after chloroform (VWR Life Sciences, USA) phase extraction according to manufacturer’s protocol. Genomic DNA was removed using a 20-minute on-column DNase I digest (Qiagen, USA). RNA concentration was quantified using Qubit 3.0 Fluorometer Broad Range RNA Assay (Life Technologies, USA). RNA integrity was evaluated using the Total RNA 6000 Pico kit on the 2100 Bioanalyzer (Agilent Technologies, USA). Mean (±SD) RNA integrity number (RIN) and 28S/18S rRNA abundance ratios were 6.81 ± 0.32 and 1.03 ± 0.27, respectively. We used Qiagen RNA*later*™ for preservation of blubber samples and isolation of RNA with RIN ≥ 7 in a previous study^14^. However, Ambion RNA*later*™ reagent used in this study did not adequately prevent RNA degradation. Due to the logistical challenges of the experiment, blubber sampling could not be repeated and we proceeded with RNA sequencing, using ribosomal RNA depletion instead of polyA mRNA enrichment during library preparation to reduce 3′ degradation bias^31^. Adequate amounts sequencing-quality RNA were obtained from 14 samples: the entire sample set (day 1 pre-ACTH, day 1 ACTH response, day 4 pre-ACTH, day 4 ACTH response) from seals 4, 6 and 7, and two samples (day 1 ACTH response, day 4 ACTH response) from seal 2.

### Library preparation and RNA sequencing

cDNA library preparation and Illumina HiSeq 4000 sequencing were conducted at the University of California, Berkeley QB3 Vincent J. Coates Genomics Sequencing Laboratory. Ribosomal RNA (rRNA) was depleted using Ribo-Zero rRNA Removal Kit (Human/Mouse/Rat; Illumina, Inc., USA). Yield and quality of mRNA were assessed using fluorometric methods and an Agilent 2100 Bioanalyzer, respectively (Agilent Technologies, USA). PrepX directional RNAseq library kits (WaferGen Bio-systems Inc., USA) were used for library preparation and libraries were quantified with KAPA Library Quantification kits for Illumina platforms (Kapa Biosystems, USA) using a Bio-Rad CFX Connect (Bio-Rad Laboratories, USA). Thirteen cycles of indexing PCR using KAPA High Fidelity Hotstart Amplification Kits (Kapa Biosystems, USA) were used before library quantification and validation. Individual libraries were barcoded, indexed as TruSeq single index per library, pooled in equimolar concentrations (400 ng), and sequenced as 100 base-pair paired-end reads on the Illumina HiSeq 4000. This generated a total of 150,055 sequenced megabases and a mean (±SD) of 26.9 ± 4.2 million paired-end 100-bp reads per sample. Sequencing data were demultiplexed and converted from bclfile to fastq file with Illumina’s bcl2Fastq software v2.18 (Illumina, USA). Raw data were uploaded to NCBI Sequence Read Archive (SRA accession: SRP157071).

### De novo transcriptome assembly

All computational analyses were conducted using the Extreme Science and Engineering Discovery Environment (XSEDE)^32^ Bridges Large High Performance Computing Cluster at the Pittsburg Supercomputing Center through allocation TG-IBN150010. Transcriptome assembly was conducted using Trinity v2.4.0^33^. Sequencing adapters and poor quality bases were trimmed from reads using Trimmomatic run in Trinity with default settings. Quality of raw and trimmed reads was evaluated using FastQC v0.11.3^34^. De novo assembly was conducted after *in silico* read normalization (50X coverage, kmer size 25 bp) using Trinity with all default settings (paired-end, strand-specific FR mode). Read mapping metrics were obtained using Bowtie2 v2.2.7^35^ and transcriptome completeness was analyzed using BUSCO v1.22^36^ (metazoan BUSCO dataset downloaded on 8/24/2017).

### Transcriptome annotation

The assembly was annotated by aligning translated DNA query sequences against the UniProtKB/SwissProt protein sequence database (downloaded on 9/29/2017; “BLASTX”) using DIAMOND v.0.8.31^37^ with e-value threshold for significant matches of 1e-3 and the “more-sensitive” option. Putative protein sequences encoded in the transcriptome were predicted using TransDecoder v3.0.1^38^ and annotated against the UniProtKB/SwissProt protein sequence database (“BLASTP”) using DIAMOND with e-value cutoff of 1e-3. Differentially expressed genes that did not have hits to the SwissProt database were manually annotated against the NCBI RefSeq database using NCBI BLASTX v2.7.1 with an e-value cutoff of 1e-5. Functional annotation of KEGG categories and GO enrichment was performed using DAVID Bioinformatics Resources v6.8^39^ with the human genome as background. Categories were considered significantly enriched at p < 0.05 (adjusted for multiple comparisons using Benjamini-Hochberg correction^40^).

### Gene expression analyses

All gene expression analyses were conducted using the Trinity pipeline. Transcript abundance was estimated using Kallisto v0.43.0^41^. Differential expression analysis was performed at the gene level using the DESeq2 package in Bioconductor v3.5^42^ in R v3.4.1 with false discovery rate cutoff of 0.05 and log2 fold-change cutoff of 1.0. Pairwise comparisons between sampling conditions are shown in Table 3. Libraries from seal 2 were included only in the ACTH response comparison (day 4 ACTH response vs day 1 ACTH response). All other comparisons were conducted using libraries from seals 4, 6, and 7 only. Protein-protein interaction network prediction was conducted using STRING v10.5^43^. Network data were imported into Cytoscape v3.5.0^44^ and filtered by experimental interactions using the edge weighted spring embedded layout. Unconnected nodes were removed from the analysis. Cytoskape network statistics were calculated using the network analyzer tool with an undirected analysis.

## Results

### Blubber transcriptome assembly

Blubber biopsies were collected from 4 juvenile northern elephant seals immediately prior to ACTH administration (“pre-ACTH”) and 4 hours following ACTH administration (“ACTH response”) on the first and fourth days of the experiment (Fig. 1). Morphometric data, ACTH doses, and corticosteroid concentrations measured at the four sampling points are presented in Table 1 (from McCormley *et al*.^12^). Fourteen cDNA libraries from day 1 pre-ACTH (seals 4, 6, and 7), day 1 ACTH response (seals 2, 4, 6, and 7), day 4 pre-ACTH (seals 4, 6, and 7), and day 4 ACTH response (seals 2, 4, 6, and 7) were sequenced using two Illumina HiSeq 4000 lanes. The libraries from seal 2 were used for assembly but were included only in the ACTH response comparison to increase power for detection of differentially expressed genes (see Methods). Raw sequencing reads were uploaded to NCBI Short Read Archive (BioProject ID: PRJNA485363, SRA accession: SRP157071). A single reference transcriptome was assembled de novo using Trinity software. We assembled 1.36 billion bases into 2,031,456 contigs (or “transcripts”) in 1,216,779 gene clusters (Table 2). The high number of transcripts is common for de novo assemblies^45^ and is likely a result of sequence polymorphism, alternative splicing, and variability in individuals’ responses to ACTH. The transcriptome assembly is available at figshare: (https://figshare.com/s/37cba07b2e9877f25b50).

**Table 1:** Experimental data for juvenile elephant seals used in the RNAseq study, including cortisol and aldosterone concentrations measured immediately before and 4 hours following administration of ACTH (pre-ACTH and ACTH response, respectively; from McCormley *et al*.^12^).

Subject	Sex	Mass (kg)	ACTH (U/kg)	Day	pre-ACTH cortisol (nM)	ACTH response cortisol (nM)	pre-ACTH aldosterone (pM)	ACTH response aldosterone (pM)
Seal 2	M	119	0.17	1 4	198.7 202.2	760.2 2626.1	812.6 1204.2	1465.8 2110.5
Seal 4	M	125	0.16	1 4	429.1 227.7	1298.5 1704.1	828.4 1476.5	2000.1 2608.5
Seal 6	F	103	0.19	1 4	357.9 494.3	1542.1 4454.3	670.9 1610.1	1251.1 2747.1
Seal 7	F	99	0.20	1 4	394.6 355.5	2598.6 2319.2	792.2 1291.3	1415.0 2123.0

ACTH: adrenocorticotropic hormone.

Aldosterone responses to the fourth ACTH administration were significantly enhanced relative to the first (p < 0.05), while cortisol responses to the first and fourth ACTH administrations did not vary in magnitude^12^.

**Table 2:** Transcriptome assembly metrics.

Metric	Value
Mean (±SD) reads per sample	26.9 ± 4.2 million
Assembled bases	1,361,692,561
Assembled contigs (transcripts)	2,031,456
Assembled transcript families	1,216,779
Annotated transcripts	372,783
Mean transcript length	670.3 bp
Read mapping rate	92.40%
Complete metazoan BUSCOs	85.8%
Complete and single-copy metazoan BUSCOs	51.6%
Complete and duplicated metazoan BUSCOs	34.1%
Fragmented metazoan BUSCOs	13.8%
Missing metazoan BUSCOs	0.4%

BUSCOs: Benchmarking Universal Single-Copy Orthologs. Read mapping rate: percent of sequenced reads mapping as proper pairs back to the assembly.

To evaluate read representation in the reference transcriptome, we mapped sequenced reads back to the assembly. Ninety-two percent of reads mapped back as proper pairs to the assembly, suggesting that it accurately represents sequenced reads (Table 2). To evaluate assembly completeness, we performed a search for metazoan Benchmarking Universal Single-Copy Orthologs (BUSCOs). Out of 978 metazoan BUSCOs in the OrthoDB catalog, 85.8% were present as complete orthologs in the seal assembly, 51.6% had matches to a single seal transcript, 34.1% were duplicated, 13.8% were fragmented, and only 0.4% were missing from the assembly (Table 2). This suggests that the blubber assembly is fairly complete as it contains the majority of orthologs predicted to be expressed in all metazoans. Due to the large number of short and potentially redundant transcripts in the assembly, we filtered the transcriptome using Transdecoder to retain only transcripts with putative protein-coding regions. The *in silico*-translated proteome contained 266,916 predicted proteins, some of which were predicted from multiple open reading frames of the same transcripts.

To identify seal homologs of vertebrate genes with known functions, the raw transcriptome assembly was annotated by translated nucleotide search (BLASTX) and the Transdecoder-predicted peptides were annotated by protein search (BLASTP) against the UniProt SwissProt protein database (e-value threshold 1e-3). BLASTX homologs were detected for 372,783 elephant seal transcripts and BLASTP homologs were detected for 164,557 Transdecoder-predicted elephant seal proteins. Transcriptome annotation data are available at figshare (https://figshare.com/s/37cba07b2e9877f25b50). To identify gene functional categories (KEGG pathways) overrepresented in the blubber transcriptome, we used the DAVID functional annotation tool. Fifty-two KEGG pathways were enriched in the assembly relative to the human genome background (adjusted p < 0.05). The largest category was metabolic pathways, which contained 882 elephant seal homologs. Other pathways of interest included the child categories purine metabolism (135 genes), insulin signaling pathway (112 genes), Wnt signaling pathway (102 genes), AMPK signaling pathway (101 genes), insulin resistance (96 genes), thyroid hormone signaling pathway (94 genes), carbon metabolism (91 genes), adipocytokine signaling pathway (58 genes), fatty acid metabolism (44 genes), and fatty acid degradation (39 genes), among others. These KEGG enrichment data were similar to those from other elephant seal blubber transcriptomes^14,46^.

### Differential gene expression

**Figure 2:**
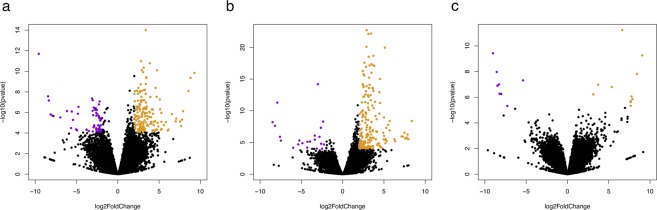
Volcano plots showing log2 fold change (x-axis) and significance (-log10 * adjusted p-value; y-axis) of genes differentially expressed during the overall ACTH response (**a**), first ACTH response (**b**), and fourth ACTH response (**c**). Significantly upregulated and downregulated genes are shown in orange and purple, respectively. Log2 fold-change and significance cutoffs for differential expression were |1| and 0.05, respectively.

**Figure 3:**
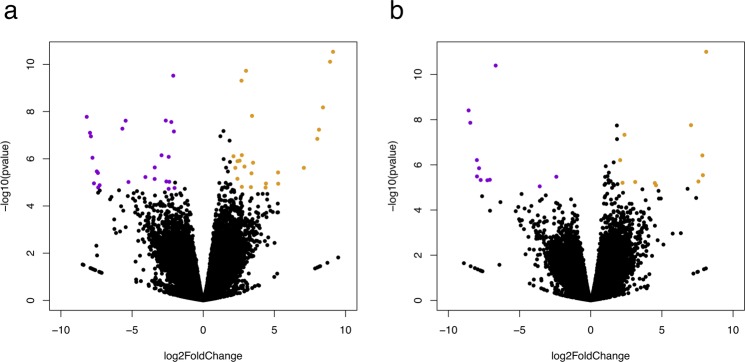
Volcano plots showing log2 fold change (x-axis) and significance (-log10 * adjusted p-value; y-axis) of genes differentially expressed in the pre-ACTH (**a**) and ACTH response (**b**) comparisons. Significantly upregulated and downregulated genes are shown in orange and purple, respectively. Log2 fold-change and significance cutoffs for differential expression were |1| and 0.05, respectively.

Differences in transcript abundance between sampling conditions were identified using DESeq2. The numbers of differentially expressed (DEG) and annotated genes (adjusted p-value < 0.05 and FDR < 0.05) identified in each pairwise comparison between conditions are shown in Table 3. The magnitude and significance of gene expression differences in each comparison are presented in Figs 2 and 3. We first examined changes in gene expression in response to the first ACTH administration, fourth ACTH administration, and over the course of the entire experiment (day 1 pre-ACTH vs. day 4 ACTH response; “overall ACTH response;” Fig. 2). We then compared expression between day 1 and day 4 pre-ACTH conditions (“pre-ACTH comparison”) and between day 1 and day 4 ACTH responses (“ACTH response comparison,” Fig. 3).

**Table 3:** Number of DEGs identified in each pairwise comparison using DESeq 2.

Comparison name	Pairwise comparison	total DEGs	upreg. DEGs	downreg. DEGs	annotated unique DEGs
First ACTH response	Day 1 ACTH response/Day 1 pre-ACTH	315	302	13	61
Fourth ACTH response	Day 4 ACTH response/Day 4 pre-ACTH	29	21	8	12
Overall ACTH response	Day 4 ACTH response/Day 1 pre-ACTH	624	479	145	99
Pre-ACTH comparison	Day 4 pre-ACTH/Day 1 pre-ACTH	66	39	27	24
ACTH response comparison	Day 4 ACTH response/Day 1 ACTH response	27	23	4	12

DEGs: differentially expressed genes. Annotated unique DEGs: differentially expressed elephant seal homologs of vertebrate proteins with known function, with multiple transcript isoforms collapsed to a single gene.

We identified 61 annotated, unique (i.e. all transcript isoforms collapsed into a single gene) DEGs in response to the first ACTH administration (Supplementary File 1), 12 DEGs in response to the fourth ACTH administration (Supplementary File 2), 99 DEGs in the overall ACTH response (Supplemetary File 3), 24 DEGs in the pre-ACTH comparison (Table 4, Supplementary File 4), and 12 DEGs in the ACTH response comparison (Table 5, Supplementary File 5). Several DEGs had hits to poorly characterized genomic regions of other mammals (first ACTH response: 31, fourth ACTH response: 7, overall ACTH response: 34, pre-ACTH comparison: 8, ACTH response comparison: 6), and thus could not provide functional insights. Functional annotation of DEG lists identified 2 Gene Ontology (GO) biological process categories enriched in first ACTH response (positive regulation of fat cell differentiation, negative regulation of transcription from RNA polymerase II promoter), 18 GO categories enriched in overall ACTH response (including fatty acid transport, long-chain fatty acid metabolic process, long-chain fatty acid-CoA ligase activity, positive regulation of fat cell differentiation, response to hypoxia, transcription factor binding) and 1 GO category enriched in the ACTH response comparison (zinc ion binding). KEGG categories were significantly enriched only in the overall ACTH response dataset (ECM-receptor interaction, focal adhesion, pathways in cancer, and small cell lung cancer).

**Table 4:** Genes differentially expressed in the pre-ACTH comparison (between pre-ACTH samples from day 1 and pre-ACTH samples from day 4).

Transcript	Log2 FC	Uniprot or NCBI Accession	Gene Name	Pathway/Process
TRINITY_DN590927_c3_g12	8.41	GCNT7_PIG	Beta-1,3-galactosyl-O-glycosyl-glycoprotein beta-1,6-N-acetylglucosaminyltransferase 7 (GCNT7)	protein glycosylation
TRINITY_DN560952_c14_g1	5.27	XP_020935075.1	6-phosphofructo-2-kinase/fructose-2,6-bisphosphatase 1 isoform X1 [Sus scrofa] (PFKFB1)	glycolysis and gluconeogenesis, response to GCs
TRINITY_DN566526_c4_g1	3.00	XM_008712174.1	PREDICTED: Ursus maritimus THAP domain containing 8 (THAP8), transcript variant X2, mRNA (THAP8)	transcription factor
TRINITY_DN590504_c4_g4	2.90	ACSM1_BOVIN	Acyl-coenzyme A synthetase ACSM1, mitochondrial precursor (ACSM1)	fatty acid activation and metabolism
TRINITY_DN560507_c9_g1	2.69	HMCS2_HUMAN	Hydroxymethylglutaryl-CoA synthase, mitochondrial precursor (HMGCS2)	ketogenesis
TRINITY_DN592323_c1_g1	2.41	TGO1_BOVIN	Transport and Golgi organization protein 1 homolog precursor (MIA3)	vesicular transport, collagen secretion
TRINITY_DN578525_c2_g2	2.13	209L2_MACMU	CD209 antigen-like protein 2 (CD209L2)	immune response
TRINITY_DN568360_c2_g1	1.91	KCRU_HUMAN	Creatine kinase U-type, mitochondrial precursor (CKMT1A)	phosphocreatine synthesis
TRINITY_DN591927_c4_g2	1.43	XM_006736873.1	cysteine dioxygenase type 1 (CDO1)	adipogenesis, taurine biosynthesis, cysteine degradation
TRINITY_DN571555_c4_g2	1.42	GPX3_RAT	Glutathione peroxidase 3 precursor (GPX3)	antioxidant response
TRINITY_DN572974_c28_g1	1.33	XM_021692420.1	adiponectin, C1Q and collagen domain containing (ADIPOQ)	fatty acid oxidation, insulin sensitivity
TRINITY_DN566880_c2_g2	1.20	HUTH_BOVIN	Histidine ammonia-lyase (HAL)	amino acid degradation
TRINITY_DN592891_c7_g1	1.13	MGST1_PIG	Microsomal glutathione S-transferase 1 (MGST1)	antioxidant response
TRINITY_DN575636_c4_g1	−1.30	ZBT46_MOUSE	Zinc finger and BTB domain-containing protein 46 (Zbtb46)	transcriptional repression, immune cell development
TRINITY_DN576776_c0_g1	−2.02	ITA2_HUMAN	Integrin alpha-2 precursor (ITGA2)	ECM organization, cell-matrix adhesion
TRINITY_DN545745_c4_g5	−2.06	FMOD_HUMAN	Fibromodulin precursor (FMOD)	collagen fibril formation
TRINITY_DN57258 _c0_g6	−2.42	HBA_ODORO	Hemoglobin subunit alpha (HBA)	oxygen transport

FC: fold change (day 4 pre-ACTH/day 1 pre-ACTH); adjusted p < 0.05.

**Table 5:** Genes differentially expressed in the ACTH response comparison (between ACTH response samples from day 1 and ACTH response samples from day 4).

Transcript ID	Log2 FC	Uniprot or NCBI Accession	Gene Name	Pathway/Process
TRINITY_DN551341_c9_g1	1.94	GLRA2_RAT	Glycine receptor subunit alpha-2 (GLRA2)	glycine receptor
TRINITY_DN570625_c1_g4	1.81	ATS16_HUMAN	A disintegrin and metalloproteinase with thrombospondin motifs 16 (ADAMTS16)	blood pressure regulation
TRINITY_DN587322_c7_g1	1.23	PIAS4_HUMAN	E3 SUMO-protein ligase PIAS4 (PIAS4)	protein sumoylation, cell stress response, metabolic homeostasis
TRINITY_DN592677_c7_g3	1.18	XM_022511147.1	Perilipin 1 (PLIN1)	lipolysis regulation, insulin sensitivity
TRINITY_DN549777_c0_g1	1.09	PLIN4_HUMAN	Perilipin-4 (PLIN4)	triacylglyceride packaging
TRINITY_DN587150_c2_g2	1.06	ZA2G_BOVIN	Zinc-alpha-2-glycoprotein (AZGP1)	lipid mobilization
TRINITY_DN591317_c4_g2	1.04	ACSL1_MOUSE	Long-chain-fatty-acid–CoA ligase 1 (ACSL1)	fatty acid catabolism, fatty acid synthesis
TRINITY_DN587426_c4_g1	1.02	CIDEA_MOUSE	Cell death activator CIDE-A (CIDEA)	lipolysis regulation, insulin sensitivity
TRINITY_DN591034_c2_g1	−1.96	BGH3_PIG	Transforming growth factor-beta-induced protein ig-h3 (TGFBI)	cell adhesion, adipogenesis
TRINITY_DN589692_c4_g2	−2.82	XM_002919434.3	PREDICTED: Ailuropoda melanoleuca C1q and tumor necrosis factor related protein 3, mRNA (C1QTNF3)	inhibition of gluconeogenesis and inflammation

FC: fold change (day 4 ACTH response/day 1 ACTH response); adjusted p < 0.05.

We next manually categorized unique annotated DEGs based on known function using literature search to understand their potential roles in repeated stress responses and metabolic homeostasis. Genes that were upregulated (n = 58) during the first ACTH response included many known GC targets and genes of interest involved in oxidative stress defenses (GPX3), fatty acid oxidation (ACADM), immune function (TLR4), adipogenesis (DKK1), lipid and monocarboxylate transport (SLC16A9), ketogenesis (HMGCS2), insulin resistance and obesity (ZBTB16), NADP biosynthesis and saturated fatty acid metabolism (NADK2), circadian rhythm (PER1), and inactive steroid receptor complexes (FKBP5). Other differentially expressed genes were associated with sphingolipid metabolism, DNA replication and repair, cytoskeleton and ECM remodeling, protein synthesis and posttranslational modification, protein transport, and embryonic development and cell differentiation. Genes downregulated (n = 3) during the first ACTH response were associated with ECM remodeling and insulin resistance (ADAMTS9), cell growth and migration (ABI2), and negative regulation of tyrosine kinase activity (PTPN12) (Supplementary File 1).

Genes upregulated (n = 10) during the fourth ACTH response included those associated with protein degradation (CACYBP), inhibition of apoptosis (DYRK3), and transcriptional regulation (NCOR1), as well as those involved in cell adhesion and migration, sphingolipid biosynthesis, and nucleotide metabolism. Genes downregulated (n = 2) during the fourth ACTH response included an activin receptor associated with adipogenesis (ACVR2A) and a protein glycosylation enzyme (Supplementary File 2). Only two DEGs (ABI2 and SPTSSB) were common between day 1 and day 4 ACTH responses.

Genes of interest that were upregulated (n = 75) during the overall ACTH response included those associated with lipolysis (CES1D), ketogenesis (HMGCS2), adipogenesis (DKK1), oxidative stress (FMO2), regulation of energy balance (LEP), fatty acid oxidation (ACSL1), fatty acid biosynthesis (ACSM1, ACSM4, SCD2, GPD1), lipid binding and transport (APOD, SLC27A6, ABCA6, ABCA10), steroid hormone transport (SLC10A6), sphingolipid metabolism (ACER2), and amino acid transport (SLC1A3). Upregulated genes also included an enzyme that links glycolytic and triglyceride synthesis pathways (GPD1), angiotensinogen (AGT) precursor, and a transcriptional activator that increases sensitivity to low GC concentrations (GMEB1). Genes of interest that were downregulated (n = 24) over the course of the entire experiment included those associated with inflammation and insulin resistance (CD44), stress and hormone responses (DRG1), local production of triiodothyronine (DIO2), regulation of glycolysis (PFKFB1), gene transcription and adipocyte differentiation (JUNB), as well as those involved in ECM remodeling and cell adhesion (Supplementary File 3).

Annotated genes upregulated (n = 13) in the pre-ACTH comparison were associated with processes of interest including glycolysis (PFKFB1), ketogenesis (HMGCS2), fatty acid activation and metabolism (ACSM1), regulation of fatty acid oxidation and insulin sensitivity (ADIPOQ), adipogenesis (CDO1), protection from oxidative damage (GPX3, MGST1), immune response (CD209L2), and amino acid degradation (HAL). Other upregulated genes were associated with phosphocreatine synthesis, collagen secretion, gene transcription, and protein glycosylation. Genes downregulated (n = 11) in the pre-ACTH comparison were associated with transcription (ZBTB46), suppression of inflammation (SETD6), oxidative stress protection (GDAP1) and transcriptional repression by nuclear receptors and inhibition of adipogenesis (NCOR1). Other downregulated genes were associated with ECM organization, cell adhesion, and motility, hematopoiesis and oxygen transport (Table 4, Supplementary File 4).

Annotated genes that were upregulated (n = 9) in the ACTH response comparison included those involved in lipid droplet formation (PLIN1, PLIN4, CIDEA), lipolysis (AZGP1), fatty acid metabolism (ACSL1), as well as cell adhesion and ECM remodeling, protein sumoylation, and glycine binding. Genes downregulated in the ACTH response comparison (n = 3) included those involved in adipogenesis, adipokine signaling, and fatty acid uptake (THBS1), lipid oxidation (C1QTNF3), and cell adhesion (TGFBI)(Table 5, Supplementary File 5).

**Figure 4:**
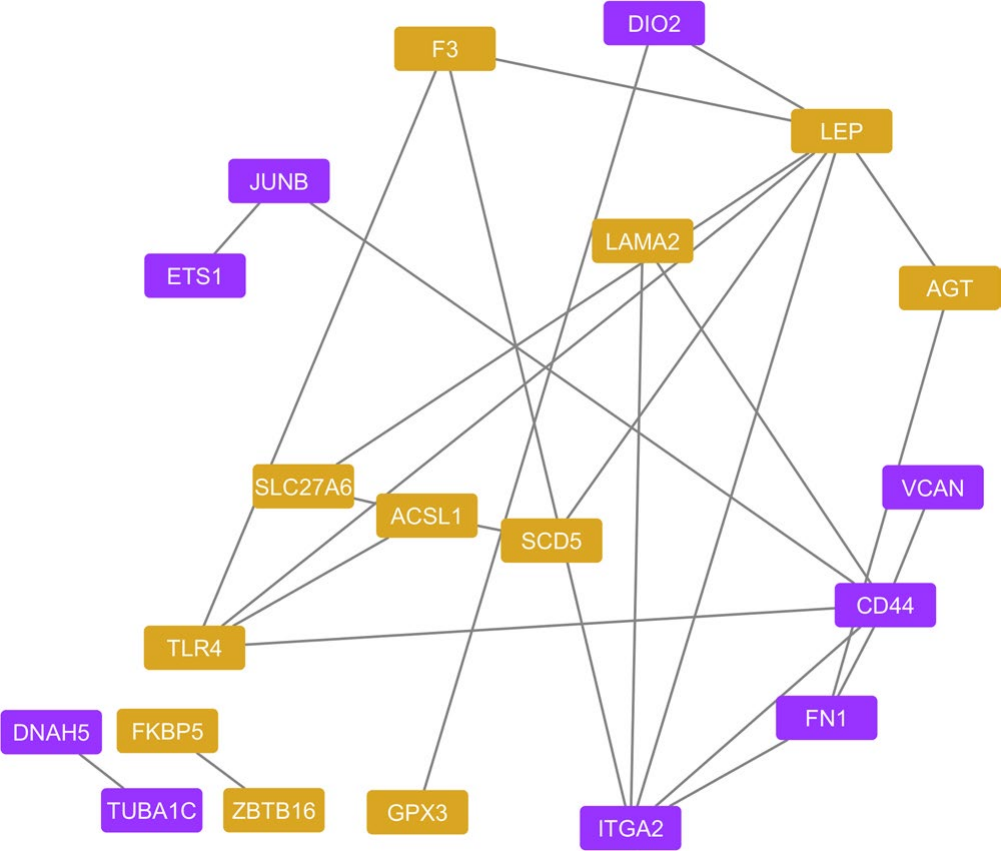
Predicted protein-protein interaction (PPI) network (p < 0.05) for genes differentially expressed over the course of the experiment (overall ACTH response). Upregulated and downregulated genes are shown in orange and purple, respectively.

Lastly, we examined the potential for interaction between proteins encoded by annotated DEGs by conducting protein-protein interaction (PPI) network analysis using the overall ACTH response dataset. The predicted PPI network contained 20 nodes, 25 edges, network density of 0.132, network heterogeneity of 0.687, network centralization of 0.263, average local clustering coefficient of 0.135, and PPI network enrichment p-value of 0.00386 (Fig. 4), suggesting that DEGs are likely functioning as a coordinated network to regulate cellular and metabolic responses to repeated ACTH administration.

## Discussion

Overall, this study shows that gene expression differences between single and repeated ACTH responses in marine mammal blubber are detectable despite similarities in cortisol responses, providing more sensitive indication of stress response states than endocrine profiles alone. We administered ACTH once daily for four days to juvenile northern elephant seals and previously reported significant elevation in corticosteroid levels in response to the first and fourth administrations^12^. However, while the aldosterone response was facilitated by repeated ACTH administration, the cortisol response was not, highlighting the unreliability of glucocorticoid measurements alone for discriminating between acute and repeated HPA axis activation. Therefore, we used RNAseq to examine downstream changes in blubber gene expression in response to repeated ACTH administration. Such changes in blubber gene activity reflect activation of genomic and cell-surface hormone receptors, including glucocorticoid receptor (GR), mineralocorticoid receptor (MR), and the adrenocorticotropic hormone receptor (MC2R) in multiple cell types, capturing the complexity of target tissue responses^47–49^. We identified DEGs during the responses to both the first and fourth ACTH administration, although the number of DEGs identified in response to the latter was much smaller than the former. We also identified transcriptional differences between pre-ACTH and post-ACTH response states from the first and fourth days. Annotated DEGs were associated with functions such as lipid and carbohydrate metabolism, adipogenesis, adipokine regulation of energy balance, insulin resistance, oxidative stress, transcriptional regulation, and ECM remodeling in other mammals and provide insights into mechanisms by which repeated HPA axis activation may affect marine mammal physiology.

Genes upregulated in response to the first ACTH challenge were associated with protection from oxidative damage, fatty acid oxidation, immune response, adipogenesis, nutrient transport, sphingolipid and ketone metabolism, cell differentiation, cytoskeletal and ECM remodeling, and insulin resistance and obesity in other animals, while downregulated genes were associated with ECM remodeling and cell migration. Several identified DEGs are primarily known for their roles in other biological processes (e.g. embryonic development) and have only recently been implicated in lipid metabolism and adipogenesis. These include GDF3 and DKK1, which promote adipogenesis and metabolic homeostasis^50,51^, and the transcriptional regulator ZBTB16, which plays a role in obesity and brown adipose energetics^52^. This is consistent with pro-adipogenic effects of GCs in other mammals^24^. Other genes upregulated in response to the first ACTH adminstration included those associated with immune response, which is consistent with studies in other mammals in which acute stress enhanced immune capacity, while chronic stress had immunosuppressive effects^53^. DKK1 and similar functional classes of genes associated with adipogenesis, immune response, and extracellular matrix function were also upregulated in blubber 2 hours after a single ACTH administration^14^. This suggests that transcriptional changes measured 2 and 4 hours after ACTH administration are capturing the same biological response in blubber. Upregulation of genes involved in lipid metabolism is consistent with known lipolytic effects of corticosteroids and the increase in circulating free fatty acid levels that has been measured in response to ACTH in this species^25,28,54^. Interestingly, one upregulated gene, FKBP5, is a GR antagonist^55^, suggesting a potential negative feedback mechanism to limit acute responses to ACTH. Genes downregulated in response to the first ACTH administration were assocated with ECM remodeling and cell migration, suggesting suppression of some energy-intensive functions during the ACTH response. However, many upregulated genes were also involved in ECM remodeling, highlighting the complexity of this specialized connective tissue.

Genes upregulated in response to the fourth ACTH administration included those associated with regulation of transcription, protein degradation, inhibition of apoptosis, and cell migration, while downregulated genes included those associated with adipogenesis and post-translational protein modification. Significantly fewer DEGs were identified in response to the fourth ACTH administration compared to the first, potentially because many cellular responses were already engaged by the previous ACTH responses. Upregulated genes included two that may protect blubber tissue from deleterious effects of repeated corticosteroid exposure: DYRK3, a kinase that phosphorylates and activates the sirtuin SIRT1 and promotes cell survival during stress^56^ and NCOR1, a nuclear corepressor that mediates ligand-induced downregulation of the GR gene^57^. DYRK3 was also upregulated in response to a single ACTH administration in a previous study^14^. Surprisingly, the type II BMP receptor ACVR2A, which promotes adipogenesis by GDF6 signaling^58^, was downregulated during the fourth ACTH response, whereas pro-adipogenic factors were upregulated in response to a single ACTH administration in this and a previous study^14^. These results suggest that the transcriptional response to a fourth sequential ACTH administration may serve to promote cell survival and limit adipogenesis and GC sensitivity. However, it is unclear whether this potential negative feedback is sufficient to prevent deleterious consequences of repeated HPA axis activation, as there were dramatic gene expression differences between the baseline state on day 1 and the ACTH response state on day 4.

The largest number of DEGs was identified in the overall ACTH response comparison, as this comparison captured all gene expression differences between pre-ACTH samples on day 1 and post-ACTH samples on day 4. Upregulated genes of interest suggest that repeated ACTH administration impacted lipid and ketone metabolism, adipogenesis, redox homeostasis, and thyroid hormone signaling. Upregulation of genes encoding leptin and the ketogenesis enzyme HMGCS2 suggests a potential increase in energy expenditure and ketone production to support metabolic demands of repeated HPA axis activation. However, leptin is also involved in modulating immune responses^59^, and its role in elephant seal metabolism is currently unclear^60^. Genes associated with lipolysis and fatty acid oxidation were upregulated concomitant with those involved in fatty acid and triglyceride synthesis (e.g. ACSM1). ACSM1 was also upregulated 24 hours after a single ACTH administration in a previous study^14^. Glucorticoid-associated lipogenesis has been described in obese humans^24,61^, and may be mediated by MR^62^. Upregulation of GPD1, which promotes triglyceride synthesis in response to excess glucose, and AGT, a component of the renin-angiotensin (RAS) system associated with obesity, suggest that repeated ACTH administration may potentially increase lipogenesis in a naturally obese and insulin-resistant mammal^30^. Downregulation of the iodothyronine deiodinase DIO2, which converts thyroxine to bioactive triiodothyronine (T3), is consistent with suppression of circulating T3 levels after repeated ACTH administration that was shown in the same animals^12^. Therefore, these data suggest that fasting-adapted mammals may simultaneously metabolize and maintain lipid stores during repeated HPA axis perturbation. However, suppression of T3 and increased lipogenesis may decrease energy expenditure and impair the ability of fasting-adapted marine mammals to participate in energetically demanding life history stages.

Our previous studies have shown that while cortisol levels returned to baseline within 24 hours of each ACTH administration^12^, changes in gene expression in response to ACTH could be detected even when cortisol was no longer elevated^14^. To identify whether transcriptional changes alone can be used to detect repeated ACTH exposure, we compared gene expression profiles in blubber samples collected immediately prior to the first and fourth ACTH administrations. Genes upregulated in pre-ACTH samples from day 4 relative to day 1 were associated with regulation of glycolysis, ketogenesis, fatty acid biosynthesis, adipokine signaling, oxidative stress, and adipogenesis. Downregulated genes were associated with genetic and epigenetic regulation of transcription, mitochondrial dynamics, and immune cell differentiation. These results suggest that recovery from repeated ACTH administration may involve metabolic and cellular adjustments such as increased glycolysis and fatty acid oxidation, synthesis of fatty acids and ketones, antioxidant responses, and increased adipogenesis. Upregulation of antioxidant enzymes GPX3 and MGST1 may serve to protect seal tissues from oxidative damage associated with exposure to GCs^63,64^. Two genes upregulated in day 4 pre-ACTH samples (24 hours after the third ACTH administration) – ACSM1 and CDO1 – were also upregulated 24 hours after a single ACTH administration in a previous study^14^. However, genes associated with ketogenesis and antioxidant defenses were not altered in response to a single ACTH challenge and may therefore serve as markers of recently experienced repeated HPA axis activation.

To identify transcriptional differences between blubber responses to single and repeated ACTH administrations, we compared the ACTH response samples from days 1 and 4. We identified nine DEGs that were upregulated and three that were downregulated in day 4 ACTH response samples compared to those from day 1, and suggest them as potential markers of repeated HPA axis stimulation in marine mammal blubber. Upregulated genes were associated with lipid droplet formation, energy homeostasis, and lipolysis, while downregulated genes were associated with adipogenesis and lipid uptake and oxidation. Three upregulated genes encoded the lipid droplet-associated proteins CIDEA and the perilipins PLIN1 and PLIN4, which are involved in regulation of lipolysis and lipid droplet expansion^65^. While GCs have been shown to upregulate genes encoding some lipid droplet proteins^66,67^, they mainly affect perilipins by inhibitory phosphorylation that facilitates lipolysis^68^. Transcriptional upregulation of perilipins in response to repeated ACTH administration may function to restrict lipolysis in an attempt to conserve lipid stores in a fasting-adapted mammal. Other genes upregulated during the fourth ACTH response compared to the first included the glycoprotein AZGP1 and glycine receptor GLRA2, which promote lipolysis^69^, ADAMTS16, which is associated with regulation of blood pressure^70^, PIAS4, which inhibits AMPK and SIRT1 (and thus derepresses lipid and protein synthesis^71,72^), and CNTNAP3, a cell adhesion protein that is a marker of brown adipocytes^73^. Downregulated genes included THBS1, which is associated with adipocyte proliferation and fatty acid uptake^74^, C1QTNF3, an adipokine that increases hepatic lipid oxidation and decreases lipid synthesis, gluconeogenesis and inflammation^75^, and TGFBI, a TGF beta family member associated with type 2 diabetes and adipogenesis^76^. While interpretation of DEG roles in ACTH responses is complicated by their pleiotropic (or poorly described) functions and the heterogeneity of cell types in blubber tissue, these data suggest that repeated ACTH administration primarily increases lipid catabolism and dysregulates lipid storage. This is consistent with significant declines in circulating triglyceride levels detected after 4 ACTH administrations^77^. Therefore, repeated HPA axis activation may impact the ability of marine mammals to maintain prolonged fasts and high levels of energy expenditure required to participate in key life history stages. For example, depletion of energy reserves by repeated stress would impact reproductive expenditure (e.g. milk production, male-male competition), decreasing fitness^78^.

To our knowledge, this study is the first to examine changes in gene expression in wild marine mammals in response to repeated ACTH administration. Despite the limitations of small sample size, high degree of individual variability in ACTH responses, and small number of ACTH administrations, we identified dozens of DEGs that were differentially expressed in response to repeated ACTH administration. Most significally, we identified gene expression differences between single and repeated ACTH responses in blubber despite similarities in cortisol responses, providing more sensitive indication of response states than endocrine profiles alone. Further work is necessary to correlate transcriptional changes with metabolic and redox profiles and validate the ability of these biomarkers to discriminate baseline stress states in marine mammals. Nevertheless, these findings provide some of the first insights into the physiological impacts of repeated HPA axis stimulation in free-ranging marine mammals, and suggest potential molecular markers of repeated stress exposure that can complement other approaches used by conservation biologists to evaluate the effects of anthropogenic activity on threatened wildlife populations.

## Supplementary information

Dataset 1

Dataset 2

Dataset 3

Dataset 4

Dataset 5

## Data Availability

Raw sequencing reads were uploaded to NCBI Short Read Archive (BioProject ID: PRJNA485363, SRA accession: SRP157071). The transcriptome assembly and annotation data are available at figshare (https://figshare.com/s/37cba07b2e9877f25b50).
